# Unconventional Protein Secretion in Brain Tumors Biology: Enlightening the Mechanisms for Tumor Survival and Progression

**DOI:** 10.3389/fcell.2022.907423

**Published:** 2022-06-15

**Authors:** Rebeca Piatniczka Iglesia, Mariana Brandão Prado, Rodrigo Nunes Alves, Maria Isabel Melo Escobar, Camila Felix de Lima Fernandes, Ailine Cibele dos Santos Fortes, Maria Clara da Silva Souza, Jacqueline Marcia Boccacino, Giovanni Cangiano, Samuel Ribeiro Soares, João Pedro Alves de Araújo, Deanna Marie Tiek, Anshika Goenka, Xiao Song, Jack Ryan Keady, Bo Hu, Shi Yuan Cheng, Marilene Hohmuth Lopes

**Affiliations:** ^1^ Laboratory of Neurobiology and Stem Cells, Department of Cell and Developmental Biology, Institute of Biomedical Sciences, University of São Paulo, São Paulo, Brazil; ^2^ The Robert H. Lurie Comprehensive Cancer Center, The Ken and Ruth Davee Department of Neurology, Lou and Jean Malnati Brain Tumor Institute at Northwestern Medicine, Northwestern University Feinberg School of Medicine, Chicago, IL, United States

**Keywords:** secretion, brain, cancer, ER stress, leaderless, glioma, glioblastoma

## Abstract

Non-canonical secretion pathways, collectively known as unconventional protein secretion (UPS), are alternative secretory mechanisms usually associated with stress-inducing conditions. UPS allows proteins that lack a signal peptide to be secreted, avoiding the conventional endoplasmic reticulum-Golgi complex secretory pathway. Molecules that generally rely on the canonical pathway to be secreted may also use the Golgi bypass, one of the unconventional routes, to reach the extracellular space. UPS studies have been increasingly growing in the literature, including its implication in the biology of several diseases. Intercellular communication between brain tumor cells and the tumor microenvironment is orchestrated by various molecules, including canonical and non-canonical secreted proteins that modulate tumor growth, proliferation, and invasion. Adult brain tumors such as gliomas, which are aggressive and fatal cancers with a dismal prognosis, could exploit UPS mechanisms to communicate with their microenvironment. Herein, we provide functional insights into the UPS machinery in the context of tumor biology, with a particular focus on the secreted proteins by alternative routes as key regulators in the maintenance of brain tumors.

## Introduction

Eukaryotic cells have developed an array of mechanisms involved in protein secretion, which plays a crucial role in cellular homeostasis and cell-to-cell communication (Sicari et al., 2019). Proteins destined for secretion to the extracellular environment are initially synthesized on ribosomes in the cytoplasm and then transported to the endoplasmic reticulum (ER) (Cavalli and Cenci, 2020) in the presence of signal peptide sequences, which have the utmost importance to direct the newly produced proteins to the ER (Rehm et al., 2001). At the beginning of protein synthesis, the 7S RNA from the signal recognition particle binds to the extremity of the polypeptide chain, which pauses the translation and transports the complex (mRNA and ribosome) to ER anchorage points (Hebert and Molinari, 2007). The translation is then restarted, and, as the polypeptide chain is extended, the chaperones that reside in the ER lumen assist the newly synthesized proteins in achieving their native conformations. Alternatively, translation can occur entirely in the cytoplasm, where after synthesis, the Sec62-Sec63 complex orchestrates protein translocation to the ER lumen along with additional chaperones (Cohen et al., 2020). In the ER, proteins may undergo modifications with the support of local chaperones when necessary, being encapsulated into transport vesicles formed by COPII and addressed to the Golgi complex (Cavalli and Cenci, 2020). Once in the Golgi apparatus, these proteins undergo additional modifications and will finally be selected for transport vesicles, which bud off from the Golgi complex. Motor proteins then carry these vesicles to fuse with different portions across the plasma membrane to release their content, which is dictated by specific destination domains (Cohen et al., 2020).

**FIGURE 1:**
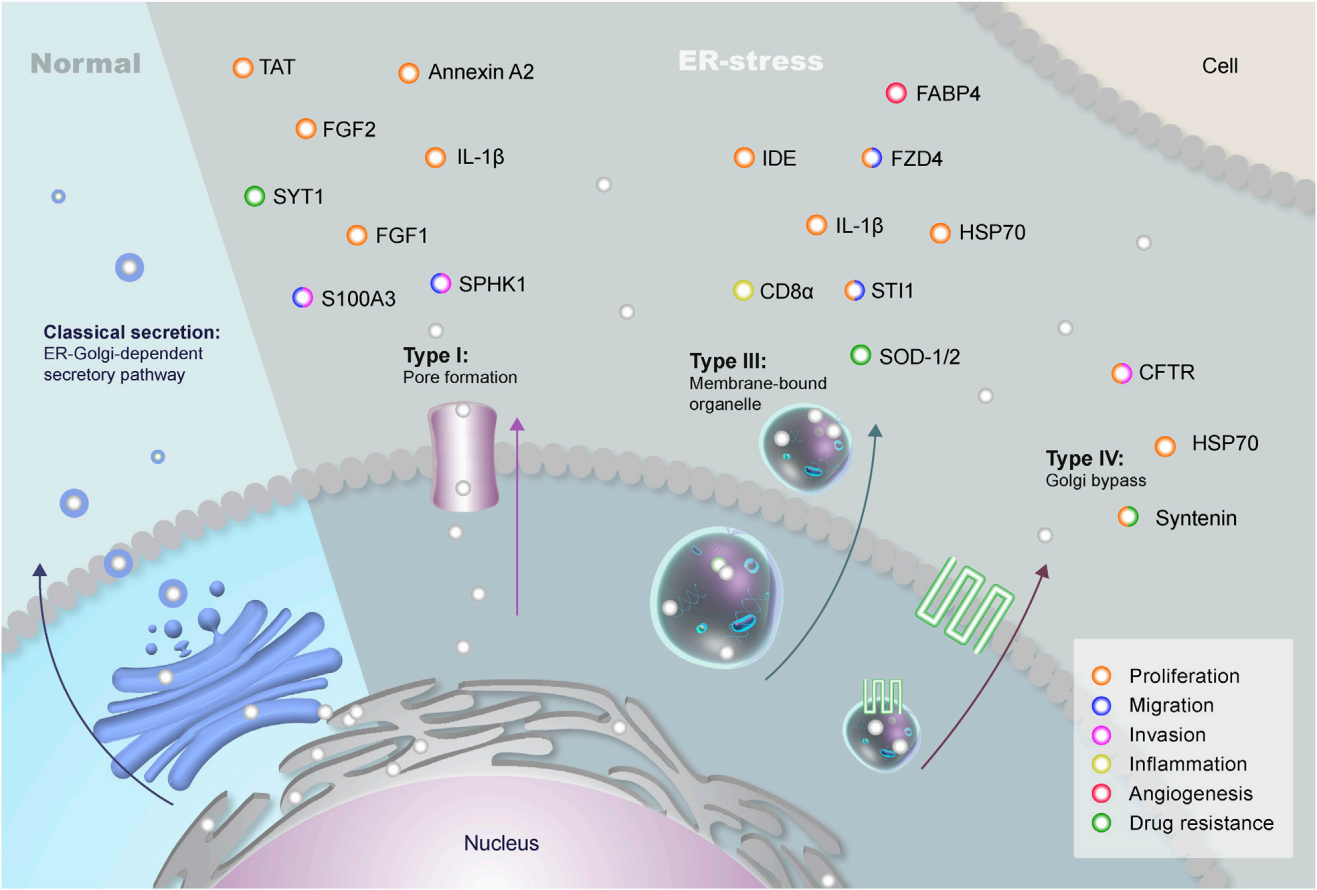
Types of Unconventional Protein Secretion in Eukaryotes. The classical secretion of proteins containing signal peptides involves the endoplasmic reticulum (ER) and the Golgi complex during normal conditions. These proteins are transported through vesicles that bud off the Golgi complex and fuse with the plasma membrane. However, leaderless proteins can be secreted through an unconventional pathway (UPS) that bypasses the Golgi during stress conditions. There are four different USPs in eukaryotes: Type I, in which proteins are secreted through a pore in the plasma membrane; Type II, with the transport of proteins through the superfamily of ATP-binding cassette (ABC) transporters (not shown in the figure); Type III, which uses autophagosomes/endosomes to transport proteins to the extracellular; and type IV, in which proteins containing a signal peptide are secreted bypassing the Golgi. Brain tumors can make use of three different types of UPS: type I (membrane pores), type III (differential vesicles), and type IV (Golgi bypass). The plethora of proteins secreted through UPS can interact with neighboring cells, promoting distinct pathways of key importance in GBM biology, such as proliferation (orange), migration (blue), invasion (pink), inflammation (yellow), angiogenesis (red) and drug resistance (green).

Therefore, the classical secretory pathway consists of the secretion of proteins containing a signal peptide and/or transmembrane domain, which leads them to the ER where COPII-coated vesicles bud to transport secretory proteins through the Golgi apparatus, reaching the plasma membrane where they are released into the extracellular milieu (Palade, 1975; Rabouille, 2017). However, during a stress response, cells present distinguished manners to express and secrete proteins to promote survival (Ferro-Novick and Brose, 2013). Under stressful conditions, the facilitated transport of proteins across the membranes of vesicles and the fast response in protein secretion along with signaling activation led to alternative pathways of secretion. It has been experimentally shown that only a limited number of proteins enter the non-classical secretory pathway (Nickel and Rabouille, 2009), including primarily fibroblast growth factors, interleukins, and galectins found in the extracellular matrix (Hughes, 1999; Nickel, 2003). These leaderless proteins lack a classical N-terminal signal peptide and function independently of the ER-Golgi network (Bendtsen et al., 2004). Additionally, their export from cells is not affected by the classical secretion inhibitors brefeldin A (BFA) (Fujiwara et al., 1988) and monensin (Schuerwegh et al., 2001; Wesche et al., 2006; Zhao et al., 2009). Recently, studies have described the cell trafficking mechanisms that avoid the conventional ER-Golgi system and comprise unconventional protein secretion (UPS) (Nickel and Rabouille, 2009; Ferro-Novick and Brose, 2013) (Figure 1). While the UPS system mainly promotes the secretion of proteins lacking the signal peptide sequences and transmembrane domains - namely leaderless proteins - it may also cause conventional proteins to be alternatively secreted via Golgi bypass (Nickel and Rabouille, 2009; Rabouille, 2017).

UPS comprises types I to IV, and the molecules secreted via non-canonical routes include cytoplasmic proteins with a central role in cell biology and its microenvironment. Briefly, type I UPS is related to the translocation of leaderless proteins across the membrane through pores. Type II is associated with ABC transporter-dependent secretion, while type III uses intracellular intermediates including endosomes, autophagosomes and lysosomes for secretion (Nickel and Rabouille, 2009; Rabouille, 2017). Finally, type IV comprises proteins that, albeit having a signal peptide or transmembrane domain bypass the Golgi apparatus, being transported from the ER to the plasma membrane. Interestingly, the family of peripheral Golgi proteins named Golgi Reassembly and Stacking Proteins (GRASPs) can participate in the Golgi bypass and in type III endosomal transport (Giuliani et al., 2011; Rabouille, 2017). These mechanisms will be better discussed through this study in a tumoral context, focusing on the role of UPS in brain tumors maintenance and progression (Figure 1).

## Unconventional Protein Secretion in Brain Tumors

Protein secretion is a fundamental process in both health and disease, playing pivotal roles in intercellular communication, which is a critical aspect in tumor progression and metastasis (Peinado et al., 2017). The tumor microenvironment (TME) is composed of blood vessels, extracellular matrix components, tumor-associated immune cells, fibroblasts, neural cells including astrocytes and neurons, and a plethora of different signaling molecules and cytokines derived from the TME (Spill et al., 2016; Greten and Grivennikov, 2019). Cancer cells require active communication with neighboring cells and the local microenvironment during tumor initiation and progression. Indeed, protein secretion has been broadly described as an essential mechanism for tumor initiation and progression, including in central nervous system (CNS) tumors such as glioblastoma (GBM) (Kucharzewska et al., 2013; Broekman et al., 2018). GBM, a grade IV astrocytoma, is an incurable malignancy and extremely aggressive neoplasm in adults characterized by microvascular proliferation, necrosis, and inter- and intratumoral heterogeneity, which may contribute to therapy resistance. Even with recent advances in GBM therapy, the overall patient survival is 15 months with few long-term survivors. Glioblastomas are characterized by presenting Isocitrate dehydrogenase (IDH) wildtype profile, usually associated with worst prognosis compared to mutant, present amplification in the epidermal growth factor receptor (EGFR), and Telomerase reverse transcriptase (TERT) promoter mutation that lead to lengthened telomeres (Louis et al., 2021). Finally, GBM also present frequently alterations in gain or loss of chromosome copy numbers (+7/−10) (Parsons et al., 2008; Louis et al., 2021). The TME exerts great influence in tumor development and secreted molecules involved in cell-to-cell communication is crucial to promoting tumor maintenance (Zhou et al., 2015). Proteins and molecules secreted by the tumor and its associated cells seem to play a crucial role in chemo and radiotherapy resistance, assisting in the poor prognosis of patients with GBM (Ou et al., 2020). It is also important to highlight that under stress conditions - such as hypoxia, which is relatively common in brain tumors - there is an increase in chemotherapy-resistant cells (Goenka et al., 2021; Singh et al., 2021). Hence, under such conditions, the tumor cells might use UPS to release proteins and molecules to modulate the TME (Figure 1).

As we will discuss in this review, the UPS routes are used by many proteins with key roles in promoting tumor chemoresistance, such as HSP70 family-like glucose-regulated protein 78 (GRP78) (Lee et al., 2008) and ATF6 (Dadey et al., 2016). Therefore, in the following sections, we will describe UPS types and address their specific roles in the context of brain tumors, focusing on the contributions of each non-canonical secretion route to tumor progression and resistance to treatment.

### Types I and II UPS—Translocation of Leaderless Proteins Through Membrane Pores

Type I UPS is characterized by the formation of plasma membrane pores that induce the translocation of cytoplasmic proteins without the participation of vesicular intermediates (Rabouille, 2017). Leaderless proteins can be translocated across the plasma membrane through pores that allow the traffic of cytoplasmic cargoes (Rabouille, 2017). Pore formation is, however, a complex process that can either be self-dependent or driven by inflammation, two pivotal mechanisms when it comes to protein release to the extracellular space (Rabouille, 2017). Regulated pore formation for UPS requires the recruitment of leaderless proteins by acidic membrane lipids at the inner leaflet of the plasma membrane, followed by oligomerization-induced membrane insertion and tyrosine phosphorylation (Rabouille, 2017). A classic example of this mechanism is the constitutive export of fibroblast growth factor 2 (FGF2). This process depends on sequential interactions of FGF2 with the phosphoinositide PI(4,5)P2 at the inner leaflet and heparan sulfate proteoglycans (HPSG) at the outer leaflet of the plasma membrane (Dimou and Nickel, 2018). Eventually, PI(4,5)P2-induced self-oligomerization stimulates membrane insertion, aided by Tec kinase-mediated phosphorylation (Steringer et al., 2015). Furthermore, FGF2 secretion is related to cell-surface ligands such as HPSG, as shown by Zehe and co-workers in a study that reported inhibition of FGF2 secretion under pharmacological inhibition of HPSG biosynthesis (Zehe et al., 2006). This data indicates that HPSG drives the translocation of FGF2 across the membrane through a molecular trap (Zehe et al., 2006). In detail, several cis-elements participate in FGF2 secretion, namely: K127/R128/K133 forming the PI(4,5)P2 binding pocket, Y81 being the target of Tec kinase, and two cysteine residues C77/C95 promoting FGF2 oligomerization, as well as four trans-acting factors: the aforementioned PI(4,5)P2, ATP1A1, Tec kinase, and HPSGs (Steringer et al., 2017). Interestingly, FGF1 and FGF2 are soluble molecules well described in the brain TME. FGF1 is a 140 amino-acid polypeptide belonging to the fibroblast growth factor family (Jaye et al., 1986; Di Serio et al., 2008) that binds to FGF receptors (FGFR), as well as other membrane receptors, such as integrin. FGF1 receptor binding stimulates a plethora of biological processes related to tumor progression, such as cell survival, proliferation, angiogenesis, differentiation, and migration (Mori et al., 2008; Yamaji et al., 2010). In brain tumors, such as gliomas, FGF1 is involved in chemotaxis and migration of tumor cells (Brockmann et al., 2003) which primarily express the FGF1B and FGF1D isoforms (Myers et al., 1995). This protein has also been considered a therapeutic target in glioma, in which the inhibition of its receptor FGFR1 decreased tumor growth (He et al., 2018).

The FGF2 is either located in the nucleus and the cytosol or released in the extracellular milieu through UPS (Akl et al., 2016). While most of its physiological functions are shared with FGF1 (Mori et al., 2008; Yamaji et al., 2010), FGF2 plays a vital role in tumor-induced angiogenesis, contributing to tumor growth. FGF2 is overexpressed in human cancers, including gliomas, and acts as an autocrine and paracrine angiogenic factor (Takahashi et al., 1992; Akl et al., 2016). In gliomas, both FGF2 and VEGF seem to have an essential role in regulating tumor growth and angiogenesis, indicating that their inhibition could be implemented as an antitumoral treatment (Bian et al., 2000). In addition, FGF2 can promote proliferation and cell survival through the activation of the Akt signaling pathway (Wang et al., 2015), which corroborates the fact that anti-FGF2 antibodies inhibited both anchorage-dependent and independent tumor growth of glioma U87MG and T98G cells (Takahashi et al., 1992). For instance, FGF2 membrane translocation through the membrane pore occurs in a fully folded conformation that requires an interaction with PIP2, which causes FGF2 to oligomerize (Torrado et al., 2009). Only then this complex can achieve membrane insertion, highlighting the need for an internal quality control mechanism that ensures the secretion of fully folded and biologically active FGF2 proteins (Torrado et al., 2009).

Interleukin-1β (IL-1β) secretion also follows the type I UPS pathway upon inflammatory cues in monocytes, macrophages, and dendritic cells (Rabouille, 2017). IL-1β is a polypeptide related to host defense and homeostasis and has been shown as one of the many mediators of infection, inflammation, and autoimmune diseases (di Giovine et al., 1991). Although IL-1β does not directly bind to PI(4,5)P2, it has been shown that inflammasome activation induces pores in the plasma membrane that allows IL-1β to reach the extracellular space (Cavalli and Cenci, 2020). Direct IL-1β secretion depends on the activation of caspase-11 in mice or caspase-4 and caspase-5 in humans, which activates gasdermin-D, a cytosolic protein containing two domains separated by a linker peptide (Kayagaki et al., 2015). Gasdermin-D undergoes a conformational change in its annular shape and drives membrane pore formation with its active amino-terminal fragment in a PI(4,5)P2-dependent manner (Liu et al., 2016). IL-1β is secreted by activated monocytes in a process related to the translocation of intracellular membranes, mostly during cell stress (Rubartelli et al., 1990). IL-1β secretion by tumor-associated macrophages in gliomas presents an essential role in tumor maintenance (Lu et al., 2020). Data from the literature demonstrate that tumor-infiltrating macrophages can help metabolism reprogramming for glioma cell survival. This effect occurs through the secretion of IL-1β since it triggers a shift in energy metabolism (from oxidative phosphorylation to aerobic glycolysis) and induces tumorigenesis and cell proliferation (Lu et al., 2020). It is noteworthy that along with tumor necrosis factor (TNF), IL-1β is one of the most critical neuro-pro-inflammatory molecules in both health and disease (Rizzo et al., 2018).

Other examples of leaderless proteins that follow the type I UPS mechanism are sphingosine kinase 1 (SPHK1), annexin A2, synaptotagmin 1 (SYT1), small calcium protein (S100A3), and TAT, among others (Rubartelli and Sitia, 1991; Kim, 2006; Rabouille, 2017; Steringer et al., 2017; Cruz-Garcia et al., 2018; Popa et al., 2018; Ye, 2018; Aliyu et al., 2019; Cavalli and Cenci, 2020; Cohen et al., 2020).

Specifically, SPHK1 is an enzyme with multiple functions, one of which catalyzes the phosphorylation of sphingosine to S1P, a lipid that regulates processes at both the intra- and extracellular levels (Wang et al., 2013). Furthermore, this enzyme is related to ceramide biosynthesis, decreasing its production, and acting as an anti-apoptotic factor (Maceyka et al., 2005). SPHK1 also regulates the inflammatory response in the nervous system due to S1P, which stimulates the TRAF2 E3 ubiquitin ligase activity and promotes the activation of the NF-κB signaling pathway (Alvarez et al., 2010; Adada et al., 2013). Loss-of-function studies have also shown that SPHK1 takes part in endocytic membrane trafficking and recycling, and is enriched in the nerve terminus, which is essential for neurotransmission (Shen et al., 2014; Lima et al., 2017). A higher expression of SPHK1 has also been shown to correlate to a poor prognosis in GBM, elevating both migration and invasion rates (Paugh et al., 2009). In addition to IL-1, EGFR, a well-described oncogenic driver in GBM, has also been described as a modulator of SPHK1 activity in glioma spheres since EGFR inhibition leads to a decrease in angiogenesis, cell viability and increases apoptosis in GBM9 cell lines (Estrada-Bernal et al., 2011), while also increasing ceramide levels (SPHK1’s precursor molecule) (Kapitonov et al., 2009; Abuhusain et al., 2013).

Moreover, annexin A2, a type I UPS protein (Rabouille, 2017), is localized to the basement membrane of epithelial cells, endothelial cells, and keratinocytes (Waisman et al., 1995), belonging to a family of calcium-dependent proteins that bind to the membrane and phospholipids (Mayer et al., 2008). In the microenvironment, annexin A2 acts as a co-receptor for plasminogen and plasminogen tissue activators, promoting vascular fibrinolysis (Seidah et al., 2012). Annexin A2 also plays an important role in cholesterol homeostasis by interacting with PCSK9, a convertase that regulates the degradation of the LDL receptor (Ly et al., 2014). In tumors, annexin A2 pseudogene 2 (A2P2) is highly expressed in tumor tissues and cell lines, indicating its potential role as a prognostic biomarker (Du et al., 2020). In addition, A2P2 inhibition in glioma cells decreased cell proliferation and aerobic glycolysis, showing a correlation with the Warburg effect in which cells shift to anaerobic glucose metabolism (Du et al., 2020). In gliomas, annexin A2 is overexpressed and associated with a mesenchymal and invasive phenotype due to its interaction with transcription factors involved in the epithelial-mesenchymal transition (EMT), such as RUNX1, FOSL2, and BHLHB2 (Kling et al., 2016; Maule et al., 2016). These data indicate the valuable role of annexin A2 as a potential therapeutic target for treating gliomas (Kling et al., 2016; Maule et al., 2016). Annexin A2 is also found in extracellular vesicles (EVs) derived from GBM cells, contributing to an increase in aggressiveness, being a direct target of microRNAs (miR) such as miR-1 and mi-R155HG (Bronisz et al., 2014; Wu et al., 2019).

SYT1 is a known gatekeeper of neurotransmitter release sensitive to calcium (Fernandez-Chacon et al., 2001), that has been marked as a differentially expressed gene in GBM and other types of human cancers, and its expression is inversely correlated with the survival of patients with cancer (Yang and Yang, 2020). In addition, this protein has also been shown to be a potential target of tumor suppressor miR-34c, which plays a key role in inhibiting cell growth and inducing apoptosis (Shi et al., 2020). On the other hand, S100A3 is a protein from the S100 family involved in epithelial cell differentiation (Kizawa et al., 2008) which has also been identified as a differentially expressed protein from grades II-IV of astrocytomas, differing according to the tumor malignancy (Camby et al., 1999). Fewer studies have also indicated that S100A3 might be related to glioma immunity, even though the mechanism is still not fully understood (Zhang et al., 2021). Lastly, TAT (or HIV-1 TAT Stimulatory Factor) is a small protein essential for HIV replication (De Marco et al., 2010) that shares a similar secretion mechanism with FGF2 (Steringer et al., 2017). Taking a closer look into the TAT’s non-conventional roles, data have shown that this molecule has a neurotoxic activity affecting cell a composite peptide containing permeabilization and membrane depolarization in neuroblastoma cells and decreasing cell growth of gliomas (Sabatier et al., 1991; Daniel et al., 2004). Interestingly, TAT (BRBP1-TAT-KLA) has also been used as a therapeutic target against metastatic brain tumor cells, inducing mitochondrial damage and apoptosis (Fu et al., 2015).

The need for alternative mechanisms of protein secretion protein secretion in cancer cells is still not fully understood. However, it might be related to cellular strategies for protein quality control, as well as to cope with the quantity and speed of protein secretion needed to respond to essential processes such as inflammation triggered by tumors. Additionally, this rapid response is very characteristic of survival mechanisms that in cancer are related to tumor progression, resistance, and recurrence processes. Despite these data describing the function of type I UPS proteins in brain tumors, the specific path of secretion of these proteins in the tumor context still requires further investigation.

Regarding type II UPS, it comprises specifically the transport through the superfamily of ATP-binding cassette (ABC) transporters, which are integral membrane proteins that bind and translocate a substrate in an ATP-dependent manner, modulating the uptake and export of macromolecules or ions (Rees et al., 2009; Wilkens, 2015; Locher, 2016; Stefan, 2019). The UPS mechanism modulated by ABC transporters was studied essentially in non-eukaryotic models. Thus, this specific model will not be further discussed in this review. However, it is noteworthy that ABC transporters are related to the unconventional secretion of heat shock Protein 70 (HSP70) in mammalian cells since they modulate the entrance of HSP70 to endolysosomal vesicles prior to secretion after the heat shock stimuli (Mambula and Calderwood, 2006; Cohen et al., 2020). The role of HSP70 in tumors is well established, and it will be further discussed in the following sections.

### Type III UPS—Vesicular Transportation of Leaderless Proteins

Type III UPS, also known as autophagosome/endosome-based secretion, is a stress-induced pathway characterized by the recruitment of membrane-bound organelles that are co-opted for secretion (Rabouille, 2017). Leaderless proteins cross the membrane of endosomes and autophagosomes and are later secreted after the organelle fuses with the cell membrane (Duran et al., 2010). Although the role of exosome-mediated secretion is well known, what might distinguish it from type III UPS is their different strategies in recruiting cargo (Ye, 2018). As an example, mammalian misfolded proteins might be secreted using type III UPS (Misfolding-Associated Protein Secretion, or MAPS), being translocated from ER to the lumen of late endosomes afterward secreted through fusion with the plasma membrane (Lee et al., 2016). In this way, there are no extracellular vesicles released. HSP70 and its co-chaperone DNAJC5 are also involved in MAPS (Xu et al., 2018). This mechanism consists of the recruitment of misfolded proteins to the surface of the ER by an associated deubiquitinase (DUB) named USP19 (Xu et al., 2018). Cargo proteins enter the lumen of late endosomes, and secretion occurs when vesicles released from endosomes fuse directly with the plasma membrane. Interestingly, this process has been associated with several key proteins in neurodegenerative diseases such as TDP-43 and α-synuclein (Fontaine et al., 2016; Lee et al., 2016).

In certain eukaryotes, type III UPS promotes the formation of Compartments for Unconventional Protein Secretion (CUPS), which were first described in yeast and are characterized by the involvement of a cup-shaped collection of tubulo-vesicular membranes that act as transport intermediates for secretion (Rabouille, 2017). The biogenesis of CUPS can be traced by the expression of the Grh1 protein (the yeast ortholog of GRASP), which migrates to distinct membrane foci of cells undergoing stress (Bruns et al., 2011) or starvation-induced autophagy (Yang and Klionsky, 2010). However, CUPS biogenesis is not triggered by rapamycin as observed in conventional pathways, and it involves proteins that are not required for classical autophagy, such as Bug1 and endosomal sorting complex required for transport (ESCRT)-II and -III (Bruns et al., 2011). CUPS can form initially from pre-existing Golgi complex membranes that mature by the contribution of endosomal membranes, depending on the activity of PI-3 kinase for its maintenance (Cruz-Garcia et al., 2014).

The involvement of autophagy-related proteins (ATG-related ATG8 and ATG9) in CUPS led to the hypothesis that a secretory and non-degrading autophagosome-like vesicle forms in UPS (Duran et al., 2010; Manjithaya et al., 2010; Bruns et al., 2011). Interestingly, ATG8-mediated autophagy in glioma cells modulates radiotherapy resistance and malignancy (Huang et al., 2017). On the other hand, ATG9A modulates an alternative lysosomal transport of ferritin in glioma cells (Goodwin et al., 2017), as well as regulates hypoxia in GBM cells, with its silencing leading to inhibition of cell proliferation and tumor growth (Abdul Rahim et al., 2017). The unconventional secretion related to autophagy was recently described in GBM modulating TMZ sensitivity through HMGB1 which, in turn, enhances M1-like polarization of tumor-associated macrophages (TAMs) (Li et al., 2022). Indeed, autophagy has been broadly studied for developing potential therapies for GBM, presenting controversial roles in the tumor’s biology since different studies have described both the induction and repression of autophagy as potential strategies for therapy (Manea and Ray, 2021).

In addition, heat shock proteins are also implicated in the transport of some cargoes in type III UPS, as transport by membrane fusion is restricted to unfolded proteins. This mechanism requires the two members of the mammalian GRASP family: GRASP55 (Dupont et al., 2011) and GRASP65 (Zhang et al., 2015), with a role for GRASP55 in the formation of secretory autophagosomes (Dupont et al., 2011).

GRASPs are comprised of a range of proteins related to Golgi reassembly and cisternae stacking. These molecules exist in homologous forms across different organisms: GRASP55 and GRASP65 in mammals; dGRASP in *Drosophila*; Grh1 in yeast; and GrpA in *Dictyostelium* (Deretic et al., 2012). The yeast GRASP Grh1 was demonstrated to colocalize with COPII in the transitional endoplasmic reticulum, and it was suggested to play roles in the early secretory process, albeit it was shown to be unessential in the organization of secretory compartments (Levi et al., 2010). In this case, the currently proposed mechanism consists of the formation of a collection of small vesicles and tubules that mature and get surrounded by flat saccules of an unknown nature that will fuse with the plasma membrane (Curwin et al., 2016). Therefore, in type III UPS, loads translocate through the membrane of the “secretory” organelle, with different structures such as a saccule, an early autophagosome, and a late endosome being reported.

Mammalian GRASP55 and GRASP65 were reported to play essential roles in the maintenance of Golgi architecture (Barr et al., 1997; Shorter et al., 1999). Despite GRASP55 and GRASP65 being homologous to each other and exhibiting similar functions, they present their own specific characteristics. The 65 kDa GRASP may be found in the cis-Golgi cistern and assembles into a complex with GM130 (a protein that has been characterized as a component and regulator of cis-Golgi structure (Nakamura et al., 1995) and p115, a membrane tethering molecule that is related to Golgi maintenance (Radulescu et al., 2011). On the other hand, the 55 KDa GRASP is localized to the medial- and trans-Golgi cisternae and does not interact significantly with the same proteins as GRASP65 (Shorter et al., 1999; Zhang et al., 2018). Since their discovery and initial characterization more than 20 years ago, GRASP55 and GRASP65 have been extensively studied by several groups. Of note, mTORC1 has been described as a phosphorylating agent of GRASP55, which consequently stacks GRASP55 within the Golgi complex (Nuchel et al., 2021). Remarkably, the lack of mTORC1 activity promotes the dephosphorylation of the GRASP protein, which, in turn, leads to a change in its localization within the cell and can consequently cause the secretion of extracellular matrix proteins via UPS (Nuchel et al., 2021). Interestingly, not only has mTORC1 surfaced as a potential therapeutic target in GBM (Ronellenfitsch et al., 2018), but studies showed that the use of mTORC1 inhibitor everolimus has great therapeutic potential against pediatric low-grade gliomas (Poore et al., 2019; Cacchione et al., 2020). GRASPs are closely related to UPS mechanisms such as type III and IV UPS (Giuliani et al., 2011), and GRASP55 is considered an unconventional secretion factor (van Ziel et al., 2019).

GRASP55 and GRASP65 have been shown to control the transport of proteins such as CD8α - a dendritic cell marker with increased expression in pro-inflammatory niches of brain tumors (Pituch et al., 2018) - and Frizzled-4 (FZD4), both containing valine residues at the C-terminal during Golgi trafficking (D'Angelo et al., 2009). In addition, proteins of the Frizzled family, such as FZD4 and FZD5, participate in the WNT signaling pathway and inflammatory processes in nervous tissue (Zhao et al., 2015) and are related to tumor initiation and cell proliferation of glioma cells (Sarkar et al., 2020), respectively, and can modulate tumor progression. Additionally, soluble Frizzled-related proteins, or sFRPs, also have an important role in glioma maintenance, modulating tumor growth and migration through MMP-2 and tyrosine phosphorylation of beta-catenin (Roth et al., 2000). Altogether, these features place Frizzled proteins as a potential therapeutic target for specific subtypes of GBM (El-Sehemy et al., 2020).

IL-1β is one of the most intensively investigated unconventional secretion loads, with several non-conventional mechanisms involved in its secretion (Andrei et al., 1999; MacKenzie et al., 2001; Brough et al., 2003; Qu et al., 2007; Lopez-Castejon and Brough, 2011). The translocation through pores was described above in this review. Moreover, when lipopolysaccharide (LPS) is the trigger, IL-1β is secreted in vesicles containing cathepsin D and Lamp-1, indicating a secretion pathway of endolysosomal origin (Andrei et al., 1999). According to this model in human monocytes, upon reaching the endolysosomes, the pro-IL-1β polypeptide is cleaved by caspase-1 and converted into a mature IL-1β protein, which is released into the extracellular space by fusion of the compartment with the plasma membrane (Piccini et al., 2008; Kimura et al., 2017). This process is mediated by the HSP90 chaperone, which interacts with a signal peptide in the mature region of IL-1β, with the participation of GRASPs, to deliver the charge to a phagophore, a precursor of the autophagosome that, when mature, transports IL-1β to the cell surface (Zhang et al., 2015). Interestingly, not only can IL-1β promote hypoxia-induced apoptosis in GBM through the inhibition of the HIF-1/AM axis (Sun et al., 2014), but it also induces tumorigenicity and promotes the formation of glioma spheres in LN-229 glioma cells (Wang et al., 2012).

The fatty acid-binding protein 4 (FABP4) is a cytoplasmic adipokine with chaperone functions whose secretion relies on UPS. Since FABP4 lacks a peptide signal sequence (Schlottmann et al., 2014), it is secreted in a GRASP-independent manner via endosomes and secretory lysosomes (Villeneuve et al., 2018). FABP4 secretion was also shown to be calcium-dependent in adipocytes (Schlottmann et al., 2014). FABP4 is upregulated in normal and low-grade gliomas, mainly related to angiogenesis (Cataltepe et al., 2012), and presents an essential role in GBM, contributing to tumor growth through the activation of WNT signaling (Li et al., 2018). FABP4 expression is observed in grade III anaplastic meningiomas, is highly expressed in vascular endothelial cells, and functions as a potential biomarker for this type of brain tumor. Additionally, other protein from the fatty acid-binding protein family, FABP7, has also been implicated as a glioma prognostic marker, and was correlated with the recurrence of several types of gliomas (Elsherbiny et al., 2013).

Like FABP4, the insulin-degrading enzyme (IDE) does not have a peptide signal sequence, relying on UPS to be transported to the extracellular space (Son et al., 2016). In HeLa cells and murine hepatocytes, IDE secretion was insensitive to inhibitors of the classical secretory pathway and conventional stimulators of protein secretion, which indicated the role of UPS in the transport and release of this protein (Zhao et al., 2009). This amyloid β protease has been investigated in Alzheimer’s disease and was shown to be secreted by astrocytes via the autophagic pathway and RAB8A, where GRASP activity was necessary for this process to occur (Son et al., 2016). Additionally, statins have been demonstrated to induce the autophagy-mediated secretion of IDE (Son et al., 2015). In N2a cells, it was shown that IDE might be transported into multivesicular bodies, which is followed by sorting into exosomes (Bulloj et al., 2010). Furthermore, the overexpression of IDE is associated with tumor progression, with its silencing inhibiting cell proliferation and promoting cell death in neuroblastoma (Tundo et al., 2013).

An interesting protein described in the literature that has been differentially secreted is the heat shock organizing protein (HOP), the human ortholog of stress-inducible protein one (STI1), which does not present a signal peptide for secretion, but it is found in the extracellular environment associated with vesicles (Hajj et al., 2013; Cruz et al., 2018). HOP is an adaptor molecule that assists the chaperones HSP70 and HSP90 in protein folding in several species, including humans (Song and Masison, 2005). Furthermore, in GBM, HOP modulates cell proliferation *in vitro* and tumor growth *in vivo* in its soluble secreted form, which interacts specifically with the cellular prion protein (PrP^C^) on the cell surface (Lopes et al., 2015; Iglesia et al., 2019). Additionally, secreted HOP binding to PrP^C^ in glioma stem-like cells (GSC) leads to an increase in self-renewal, proliferation, and migration (Iglesia et al., 2017), and the blockage of this interaction has presented a therapeutic potential in some studies (Lopes et al., 2015; Iglesia et al., 2017).

Superoxide scavenger enzyme or superoxide dismutase 1 (SOD1) is another protein that does not have a signal sequence but shows a conserved diacidic motif that determines its UPS fate (Cruz-Garcia et al., 2017). Pathologically, this motif is also present in a mutated form of SOD1 that is related to amyotrophic lateral sclerosis (Cruz-Garcia et al., 2017). SOD2, a second family member, was related to resistance to temozolomide (TMZ) in GSCs and GBM recurrence (Chien et al., 2019). In brain tumors, recombinant SOD1 and two associated with manganese (r-hMnSOD) exhibit a therapeutic potential since they can attenuate edemas by combating the oxygen-free radicals produced during the inflammatory response (Shoshan and Siegal, 1996). Indeed, the expression of several SODs and other antioxidants are inversely correlated with glioma malignancy and prognosis (Aggarwal et al., 2006), presenting low activity in tumors compared to normal tissues (Popov et al., 2003), thus supporting their anti-tumor activity. Furthermore, the transcription factor SP1 was shown to regulate SOD2 expression, which is related to TMZ resistance and recurrence in an MGMT-independent manner (Chang et al., 2017).

It is noteworthy that many leaderless proteins in the brain tumor context are related to cell survival, especially regulated by stress response regulators such as chaperones and associated molecules, inflammatory response, antioxidants, and proteins that participate in autophagy, which support the participation of UPS mechanisms in tumor progression and resistance to therapy.

### Type IV UPS—Golgi Bypass

While leaderless proteins can be secreted via unconventional routes, proteins with a signal peptide and/or a transmembrane domain can also deviate from the conventional secretory pathway. If these proteins are not directed to the Golgi apparatus on their way to vesicular organelles, the plasma membrane, or the extracellular environment, they undergo UPS via Golgi bypass, whose mechanism harbors many similarities with the other UPS types, despite certain exclusive features (Grieve and Rabouille, 2011; Rabouille, 2017). Importantly, the Golgi bypass has been a research topic of increasing interest that remains poorly understood. Although several studies point to type IV UPS being triggered by stress (ER and mechanical) (Giuliani et al., 2011), emerging evidence shows that different proteins can be constitutively secreted by both the conventional mechanism and Golgi bypass (Baldwin and Ostergaard, 2002).

The first example of proteins “skipping” the Golgi comes from a study in 1980 by Bergfeld et al., who observed this phenomenon in the formation of storage protein bodies and accumulation of proteins in the vacuole of *Sinapis alba* through electron microscopy (Bergfeld et al., 1980). Since then, the process has been observed in different organisms, including plants, fungi, *Drosophila*, and mammalian cells (Bergfeld et al., 1980; Morre, 1981; Sluiman, 1984; Schotman et al., 2008; Davis et al., 2016; Ng and Tang, 2016; Dimou et al., 2020)), indicating that this process is a conserved mechanism throughout evolution. Furthermore, the Golgi apparatus is the central organelle for protein processing, in which many resident proteases change protein composition through post-translational modification (Kulkarni-Gosavi et al., 2019; Frappaolo et al., 2020). If proteins bypass the Golgi, their structural composition is maintained as it was initially synthesized in the ER. These proteins will present the commonly high-mannose oligosaccharide N-linked core but will not be processed in Golgi, where sugar would be added to this core by resident proteases (Roth, 2002; Ito and Takeda, 2012; Fujikawa et al., 2016). Therefore, the Golgi bypass could represent a mechanism that modulates protein composition, function, and affinity with other molecules through its structural composition (i.e., glycosylation state).

Proteins that undergo the Golgi bypass can have different functions (Baldwin and Ostergaard, 2002; Gonzalez et al., 2018; Witzgall, 2018; Van Krieken et al., 2021), but all of these proteins show similar characteristics that are utilized for their identification (Grieve and Rabouille, 2011), such as resistance to BFA, which inhibits the formation of COPI coats in Golgi membranes through Arf1 activation (Zeghouf et al., 2005; Langhans et al., 2007). Thus, only proteins sorted to the Golgi bypass, and consequently do not require COPI or COPII-coated vesicles to reach the plasma membrane or the extracellular medium, are BFA-resistant (Rabouille et al., 2012). Proteins are also found to be independent of specific SNAREs involved in the ER to Golgi transport and beyond (Yoo et al., 2002). Specifically, Syntaxin 5 (STX5) is known to be extremely important to Golgi transport (Dascher et al., 1994), and protein secretion in its absence suggests the independence of these groups of proteins to reach their proper localization (Grieve and Rabouille, 2011; Kim et al., 2016). Furthermore, SNAREs are quite relevant to the biology of brain tumors. For example, Syntaxin 1 (STX1) expression supports tumor growth and invasiveness in GBM models (Ulloa et al., 2015), and several genes from the SNARE family are enriched in pediatric medulloblastoma (Huang et al., 2020b). Thus, the correlation of SNARE-independent transport with brain tumors warrants further investigation.

Another important aspect is that proteins that can bypass the Golgi appear to have one or more Postsynaptic density-95, disks-large, and zonula occludens-1 (PDZ) domains, a protein interaction module responsible for target recognition (Gee et al., 2011; Vinke et al., 2011; Liu and Fuentes, 2019). Previous studies described some of these molecules related to brain tumors, although their secretion mechanism is not fully understood. For example, the scaffold protein called syntenin, which contains two postsynaptic density protein-95/discs-large/PDZ domains, also presents as a potential new therapeutic target in GBM (Haugaard-Kedstrom et al., 2021). The highly selective inhibitor of syntenin KSL-128114 can bind to the PDZ1 domain of syntenin and demonstrates a decrease in cell viability of primary GBM cells and significantly increases survival in patient-derived xenograft mouse models (Haugaard-Kedstrom et al., 2021). Additionally, specific inhibition of syntenin activity by the PDZ1 inhibitor decreases radioresistance of human GBM cells and decreases invasion post-radiotherapy (Kegelman et al., 2017). Indeed, syntenin is a scaffold protein that acts at the cell surface, and its expression is more evident in high-grade gliomas compared to its counterparts. Syntenin also increases cell migration and invasion, and its silencing decreases tumor growth and therapy resistance (Kegelman et al., 2014; Kegelman et al., 2017). The transcriptional coactivator with PDZ-binding motif (TAZ) participates in the Hippo pathway and modulates glioma cell EMT, proliferation, invasion, differentiation, and patient survival (Bhat et al., 2011; Li et al., 2016). Other examples of PDZ-containing proteins that are essential for brain tumor biology include the Tax-interacting protein (TIP)-1 related to GBM motility (Wang et al., 2014), membrane-associated guanylate kinase inverted 3 (MAGI3), and Protein interacting with C kinase 1 (PICK1), which are inversely correlated with glioma malignancy and progression (Cockbill et al., 2015; Ma et al., 2015). However, the specific mechanisms of translocation of these proteins to the membrane of brain tumors have not been fully explored, and more research is required to confirm their association with the UPS.

The Golgi bypass could be a strategy for cells to deliver proteins to the plasma membrane and extracellular space faster than the canonical secretory pathway (Baldwin and Ostergaard, 2002; Grieve and Rabouille, 2011). The first sorting mechanism described for the Golgi bypass was discovered by observing the secretion of the cystic fibrosis transmembrane conductance regulator (CFTR). Mutated CFTR is known for its role in cystic fibrosis disease, and its most common mutation is associated with its cell surface expression (Elborn, 2016). Despite wild-type CFTR being conventionally secreted from ER exit sites using COPII-coated vesicles, wild-type and mutated CFTR also present unconventional secretion mediated by GRASP55 (Gee et al., 2011). GRASP55 can form a homodimer through their PDZ domains in the Golgi, which is important for Golgi structural assembly (Wu et al., 2020). Upon ER stress, GRASP55 is phosphorylated at serine 441 residue by a yet unidentified kinase, leading GRASP55 back to the ER as a monomer (Kim et al., 2016). Monomeric GRASP55, *via* its PDZ domain, can recognize other PDZ domains of proteins that undergo the Golgi bypass (Gee et al., 2011; Kim et al., 2016). Mouse models carrying mutations in the CFTR promoter develop ependymoma tumors and hydrocephalus, with no other alterations in vital organs such as the lungs and pancreas (Perraud et al., 1992). On the other hand, the expression of CFTR in human GBM cells is less evident when compared to normal tissue, and it abrogates GBM cell proliferation and invasion through the inhibition of the JAK2/STAT3 signaling pathway (Zhong et al., 2019). This demonstrates that the mutated CFTR may present an opposite role to its wild-type counterpart in tumors, thus suggesting a role for UPS in this process.

More recently, additional sorting machinery was proposed involving HSP70, a protein that is an essential molecular chaperone in health and disease and displays constitutive expression despite being highly induced by different stress stimuli (Rosenzweig et al., 2019). Additionally, the HSP70 family and other chaperones present significant participation in brain tumor biology, including GBMs (Iglesia et al., 2019). In the context of ER stress and UPS activation, the heat shock cognate Hsc70 (a constitutive human isoform of HSP70) associated with its co-chaperone DNAJC14 directly interacts with cargo proteins selected to the Golgi bypass, directing the cargo to the plasma membrane instead of directing it to refold or to the ER-associated degradation (ERAD) system (Jung et al., 2016). Furthermore, Hsc70 is highly expressed in tumor tissues, including gliomas, and is directly related to the poor prognosis of high-grade gliomas (HGG), where its silencing decreases tumor proliferation and survival (Sun et al., 2019).

Interestingly, in insulin-positive alpha and beta cells of patients with and without type 1 diabetes, PrP^C^ was found in the plasma membrane and the ER but not in Golgi, possibly indicating UPS by the Golgi bypass. In this work, the authors suggest that the PrP^C^’s Golgi bypass observed in the human pancreas could be through HSP70/DNAJC14 or GRASP55 (Hiller et al., 2021). As aforementioned, PrP^C^ associates with the HSP70/90 co-chaperone STI1/HOP in the cell surface (Lopes et al., 2005; Rosenzweig et al., 2019), which could indicate a greater tendency of secretion to be via HSP70/DNAJC14, although this hypothesis must be tested and the mechanism for PrP^C^ UPS needs to be clarified. As previously mentioned, the interaction of PrP^C^ and STI1 in GBM cells promotes the self-renewal and migration of GSCs, as well as proliferation and survival (Iglesia et al., 2017) of heterogeneous tumors (Lopes et al., 2015).

In addition, the HSP70 co-chaperone, heat shock protein 70-binding protein (HspBP), is usually found overexpressed in brain tumors and presents diverse cellular sub-localizations, including in the extracellular media when compared to normal tissue (Graner et al., 2009). Furthermore, HspBP interacts with several members of the HSP70 family-like glucose-regulated proteins 75 and 78 (GRP75 and GRP78, respectively) and Hsp110, among others, including cell surface receptors. However, in normal conditions, HspBP binds only Hsc70, GRP75, and HSP110 (Graner et al., 2009), demonstrating a different stress response in tumor conditions that includes its secretion. Notably, GRP78 was associated with ER stress in another mechanism broadly described in the literature, called the unfolded protein response (UPR) (Markouli et al., 2020).

The UPR consists of an adaptive response to ER stress usually caused by by the accumulation of unfolded proteins (Le Reste et al., 2016). This mechanism involves the inhibition of broad protein translation while increasing the translation of chaperones to enhance the folding capacity and the degradation of unfolded proteins to clear the ER (Mann and Hendershot, 2006). A single chaperone, GRP78, controls these processes. GRP78 acts through the release of its binding to three proteins: Activating Transcription Factor 6α (ATF6) (Haze et al., 1999), Inositol Requiring Enzyme 1 (IRE1α) (Tirasophon et al., 1998), and PKR-like endoplasmic reticulum kinase (PERK) (Harding et al., 1999). Once GRP78 dissociates from the binding proteins, it associates with the hydrophobic domains of unfolded proteins, leading to the phosphorylation of the primary binding proteins and consequent activation of signaling to mediate the stress response. Moreover, ATF6 modulates the transcription of genes related to protein folding and ERAD. IRE1α also modulates protein folding and ERAD, lipid synthesis and secretion, and PERK mediates amino acid metabolism, folding, autophagy processes, and apoptosis (Bertolotti et al., 2000; Acosta-Alvear et al., 2007; Yamamoto et al., 2007; Hetz et al., 2009; Scriven et al., 2009; Ye and Koumenis, 2009; Chevet et al., 2015; Dejeans et al., 2015). It is broadly discussed in the literature that tumors secrete specific cores of molecules to promote angiogenesis, proliferation, invasion, survival, and even reprogramming and EMT (Le Reste et al., 2016; Markouli et al., 2020). Since UPR mechanisms can remodel the cascade of activated signaling to respond to ER stress, it is natural to associate this process with the ER stress-mediated UPS.

Several studies associate ER stress and the central molecules of UPR modulation, ATF6, IRE1α, and PERK with brain tumor biology (Markouli et al., 2020). For example, ATF6 was associated with GBM resistance to radiotherapy (Dadey et al., 2016) and the formation of a pro-angiogenic GBM TME since it responds to VEGF secretion (Karali et al., 2014). ATF6 signaling was described as modulating NOTCH signaling in gliomas in hypoxia conditions, leading to radiotherapy resistance of GSCs (Dadey et al., 2016). In meningiomas, ATF6 expression levels were associated with tumor aggressiveness (Iglesias Gomez and Mosquera Orgueira, 2014). IRE1α was related to glioma growth, angiogenesis, and invasion (Drogat et al., 2007; Dejeans et al., 2012; Auf et al., 2013; Pluquet et al., 2013; Jabouille et al., 2015; Minchenko et al., 2020). Gliomas expressing low levels of IRE1α present impaired growth and angiogenesis ability and increased survival of glioma xenograft-bearing animals (Auf et al., 2010). IRE1α can also modulate the expression of hypoxia-related genes in GBM (Minchenko et al., 2016), hypoxia-induced cell death (Romero-Ramirez et al., 2004; Minchenko, et al., 2020), and the neuroinflammation associated with gliomas through the secretion of interleukins and activation of NF-κB (Hu et al., 2006; Auf et al., 2010). IRE1α activation in ER stress of gliomas caused by nutrient starvation or hypoxia leads to VEGF-mediated angiogenesis (Drogat et al., 2007), and IRE1α signaling activation was correlated with the increase of invasion markers expression and tumor infiltration by immune cells (Lhomond et al., 2018).

PERK is related to tumor metabolism and therapy resistance of GBM (Hamed et al., 2010; Yacoub et al., 2010; Hou et al., 2015). Indeed, gliomas do present high levels of glycolysis, also due to the hypoxia, which supports tumor growth, and this mechanism may be regulated by PERK and the activation of Akt signaling (Hou et al., 2015). The inhibition of upstream effectors of PERK sensitizes GSCs to radiotherapy and decreases recurrence (Yang et al., 2020). Furthermore, PERK modulates angiogenesis in GBM in hypoxic conditions (Soni et al., 2020), and it is correlated with the stem-like cell phenotype through the modulation of SOX2 expression (Penaranda-Fajardo et al., 2019). In medulloblastomas, PERK activation is associated with cerebellar dysplasia (Lin et al., 2011), angiogenesis, cell migration (Jamison et al., 2015), and tumorigenesis (Ho et al., 2016).

Furthermore, GRP78 is highly expressed in gliomas, assisting tumor initiation and protection against cell damage and death mediated by reactive oxygen species (Suyama et al., 2014). In GBM, this protein is also overexpressed, especially in recurrent GBM, and correlates with tumor progression (Wen et al., 2020) and therapy resistance to TMZ (Pyrko et al., 2007; Lee et al., 2008) and radiation (Lee et al., 2008; Dadey et al., 2016). GRP78 expression is increased in endothelial cells derived from clinical gliomas as compared to endothelial cells from healthy tissues. Interestingly, these patient gliomas-derived endothelial cells are highly resistant to apoptosis, and GRP78 expression in these cells was recently associated with the resistance to chemotherapist agents (Virrey et al., 2008). The expression of GRP78 was evaluated in GBM treated with the UPR inducer TAK-243, a ubiquitin-activating enzyme 1 (UBA1) inhibitor, to inhibit tumor cell viability and, interestingly, the expression of GRP78 was related to the stem-like phenotype and increased sensitivity of these cells to the treatment (Liu et al., 2021). Another enzyme, the Ubiquitin-conjugating enzyme E2T (UBE2T), is correlated with tumor recurrence, highly expressed in GBM, and associated with poor prognosis, EMT regulation, and invasion of GBM cells through GRP78 (Huang et al., 2020a). Also, in recurrent GBM, it was demonstrated that overexpression of GRP78 in patient-derived samples correlated with poor survival and tumor progression (Dadey et al., 2016). Data from the literature demonstrated that a recurrent glioma sample that was subjected to the Stupp protocol, which consists of a combination of TMZ with fractionated radiation, presented a higher level of GRP78 compared to primary samples and was correlated to ER stress and therapy resistance (Shah et al., 2019). Regarding therapeutic possibilities using ER-stress as a target against brain tumors, the treatment with betulinic acid (BA) inhibited GBM primary and recurrent tumor cells growth through the activation of UPR by the PERK axis (Lo et al., 2020).

Indeed, therapeutic possibilities have been studied using ER stress as a target against brain tumors. For example, the use of ursodeoxycholic acid (UDCA) alone or associated with the proteasome inhibitor bortezomib (BTZ) leads to G1 cell cycle arrest and consequent decrease in cell viability by apoptosis in GBM, triggering ER stress through the ATF6-IRE1-PERK axis (Yao et al., 2020). Another example is the combination of TMZ with Fluoxetine (FLT), which activates ER stress through the ATF6-IRE1α-PERK cascade, causing an increase in early apoptosis levels and inhibition of cell proliferation in glioma (Ma et al., 2016). The combination treatment of TMZ and simvastatin (Simva) also effectively triggers UPR and leads to apoptosis. The use of inhibitors such as MKC8866 (IRE) and GSK-2606414 (PERKi) led to an impairment in the viability of GBM cells (Dastghaib et al., 2020; Le Reste et al., 2020). Additionally, the stimulation of UPR with 2-Deoxy-D-Glucose (2-DG) enhanced the radiotherapy effects in GSCs by increasing apoptosis (Shah et al., 2019).

## Conclusion and Future Perspectives

Herein, we described the mechanisms of UPS and their participation in brain tumor maintenance. The UPS system is related to survival mechanisms since it allows the activation of alternative paths that promote the stress response and rapid turnover of cell behavior, either through the secretion of leaderless proteins or the fast release of proteins across the membrane, some bypassing the Golgi. On the other hand, the biology of cancer cells are remarkable, given that they present an outstanding ability to survive and proliferate in adverse environments. Some of these behaviors are sustained by substantial expression and secretion of factors related to stress response by those cells, as their microenvironment is enriched in and has a high activation of multiple signaling pathways (Rabouille, 2017; Dimou and Nickel, 2018) (Supplementary Table S1).

In this context, the UPS system can actively promote cancer survival and response to the TME, including the ability of the cells to resist therapy. Brain tumors are highly lethal and present several attributes that compromise treatment efficacy, such as the location of the tumor, the invasive capacity, therapy resistance, and quiescence ability. It is widely described in the literature that the role of the TME in the survival of brain tumors, and many secreted proteins, autocrine or paracrine, were correlated with key features related to the prognostic of patients with brain tumors (Quail and Joyce, 2017). Furthermore, the recent identification of UPS mechanisms and their study could bring together the significant correlation of non-canonical protein secretion with cancer cell survival and present a new field of study for therapy development. Indeed, the hypothesis of non-canonical pathways of secretion assisting tumor evasion override and overtake the options for inhibitors targeting classical secretion pathways. Nevertheless, very little is currently understood about the regulation of UPS in brain tumors, as this is a new and emerging research subject. A greater comprehension of the mechanisms underlying the processes involved in the activation and maintenance of UPS pathways is essential for developing new inhibitory drugs for the treatment of brain tumors and the advancement of cancer therapeutics.
