# Supplementary material for: Unconventional Protein Secretion in Brain Tumors Biology: Enlightening the Mechanisms for Tumor Survival and Progression
Source: Front Cell Dev Biol. 2022 Jun 15;10:907423. doi: 10.3389/fcell.2022.907423 (PMC9242006; doi:10.3389/fcell.2022.907423)
Supplement: Supplementary file 1 [file Table1.DOCX]

| **Protein** | **UPS** | **Physiological role** | **Role in brain tumors** |
| --- | --- | --- | --- |
| FGF1 | I | Fibroblast growth factor essential in cell survival, division, angiogenesis, differentiation and migration (Mori et al., 2008; Yamaji et al., 2010) | Involved in chemotaxis and tumor migration in GBM cell lines (Brockmann et al., 2003) |
| FGF2 | I | Fibroblast growth factor essential in cell survival, division, angiogenesis, differentiation and migration (Mori et al., 2008; Yamaji et al., 2010). | Regulates tumor growth and angiogenesis in astrocytomas (Bian et al., 2000); Promotes proliferation and cell survival in GBM (Wang et al., 2015). |
| IL-1β | I, III | Polypeptide related with host defense and homoeostasis. Mediator of infection, inflammation and autoimmune diseases (di Giovine et al., 1991). | Its secretion by tumor-associated macrophages in glioma is related to tumor maintenance, proliferation, and survival (Lu et al., 2020). |
| SPHK1 | I | Catalyzes the phosphorylation of sphingosine to S1P (Wang et al., 2013). Related to ceramide biosynthesis (Maceyka et al., 2005) and regulates the inflammatory response in the nervous system due to S1P (Adada et al., 2013; Alvarez et al., 2010). | Correlates to a poor prognosis in GBM elevating both the migration and invasion rates, possibly due to the secretion of IL-1 (Paugh et al., 2009). |
| Annexin A2 | I | Calcium-dependent proteins that bind to the membrane and phospholipids (Mayer et al., 2008). Acts as a co-receptor for plasminogen and plasminogen tissue activator, promoting vascular fibrinolysis (Seidah et al., 2012). | Its inhibition in glioma cells decreased cell proliferation and aerobic glycolysis (Du et al., 2020). Overexpressed in GBM associated with EMT (Kling et al., 2016; Maule et al., 2016). |
| SYT1 | I | Gatekeeper of neurotransmitter release sensitive to calcium (Fernandez-Chacon et al., 2001). | Differentially expressed genes in GBM, inversely correlated with survival and good prognosis (Yang and Yang, 2020). |
| S100A3 | I | Involved in epithelial cell differentiation (Kizawa et al., 2008). | Differentially expressed protein from grades II-IV of astrocytomas, differing according to the tumor malignancy (Camby et al., 1999). |
| TAT | I | Essential protein for HIV replication (De Marco et al., 2010). | Neurotoxic activity decreasing cell growth in several types of gliomas (Daniel et al., 2004; Sabatier et al., 1991). |
| HSP70 | II, III, IV | Essential molecular chaperone in health and disease (Rosenzweig et al., 2019). | Highly expressed and related to high grades of gliomas' poor prognosis, and its silencing decreases tumor proliferation and survival (Sun et al., 2019). |
| CD8α | III | Dendritic cell marker (Pituch et al., 2018). | Highly expressed in pro inflammatory niches of brain tumors (Pituch et al., 2018). |
| FZD-4/5 | III | Participates in the WNT signaling pathway and inflammatory processes in nervous tissue (Zhao et al., 2015). | Related with stem-like and cell proliferation of brain cancer cells (Sarkar et al., 2020), modulating tumor progression and presenting therapeutic potential in GBM (El-Sehemy et al., 2020). |
| FABP4 | III | Cytoplasmic adipokine with chaperone functions that has been emerging as a metabolic and heart disease biomarker (Schlottmann et al., 2014; Villeneuve et al., 2018). | Upregulated in normal and low-grade glioma tissues, being mainly related to angiogenesis (Cataltepe et al., 2012). Presents an essential role in GBMs, contributing to tumor growth through the activation of WNT signaling (Li et al., 2018). |
| IDE | III | Enzyme that degrades insulin (Son et al., 2016). | Its silencing leads to a decrease in tumor proliferation and an increase in cell death (Villeneuve et al., 2018). |
| SOD-1/2 | III | Superoxide scavenger enzyme (Leinartaite and Johansson, 2013). | Its expression is inversely correlated with tumor malignancy and prognostic (Aggarwal et al., 2006), presenting low activity in tumors compared to normal tissues (Popov et al., 2003). |
| STI-1 | III | Adaptor molecule that assists the chaperones HSP70 and HSP90 in protein folding (Song and Masison, 2005). | Modulates cell proliferation in vitro and tumor growth in vivo (Lopes et al., 2015) and leads to an increase in self-renewal, proliferation, and migration due to its interaction with PrP^C^ (Iglesia et al., 2017). |
| Syntenin | IV | Scaffold protein (Kegelman et al., 2014; Kegelman et al., 2017). | Increases cell migration and invasion, and its silencing decreases tumor growth and therapy resistance (Kegelman et al., 2014; Kegelman et al., 2017). |
| CFTR | IV | Mutated isoform known for its role in cystic fibrosis disease (Elborn, 2016). | Its expression in human GBM lines is less evident when compared to normal tissue and abrogates GBM proliferation and invasion through the inhibition of the JAK2/STAT3 signaling pathway (Zhong et al., 2019). |
